# Targeting tumor-associated macrophages to overcome immune checkpoint inhibitor resistance in hepatocellular carcinoma

**DOI:** 10.1186/s13046-025-03490-9

**Published:** 2025-08-05

**Authors:** Fen Liu, Xianying Li, Yiming Zhang, Shan Ge, Zhan Shi, Qingbin Liu, Shulong Jiang

**Affiliations:** 1 College of Traditional Chinese Medicine, Shandong University of Traditional Chinese Medicine, Jinan, 250000 China; 2 Clinical Medical Laboratory Center, Jining First People’s Hospital, Shandong First Medical University, Jining, 272000 China; 3 Hepatobiliary Surgery Department, Jining First People’s Hospital, Shandong First Medical University, Jining, 272000 China; 4 College of First Clinical Medicine, Shandong University of Traditional Chinese Medicine, Jinan, 250000 China; 5 Institute of Basic Research in Clinical Medicine, China Academy of Chinese Medical Sciences, Beijing, 100700 China

**Keywords:** Hepatocellular carcinoma, Immunotherapy, Immune checkpoint inhibitors resistance, Tumor-associated macrophages, Tumor immune microenvironment

## Abstract

Hepatocellular carcinoma (HCC) remains a critical global health concern, particularly in regions with high endemicity of hepatitis B, hepatitis C, and non-alcoholic fatty liver disease. Immunotherapy, particularly immune checkpoint inhibitors (ICIs), has emerged as a promising therapeutic strategy for advanced HCC. Despite encouraging results, primary and acquired resistance to ICIs continues to pose significant challenges in clinical practice. Recent research has identified tumor-associated macrophages (TAMs) as key contributors to immune evasion and ICI resistance in HCC, primarily through polarization to the M2 phenotype. M2-polarized TAMs secrete a range of immunosuppressive cytokines that inhibit T cell activation and promote tumor progression through processes such as angiogenesis and epithelial-mesenchymal transition. These mechanisms compromise the efficacy of ICIs and facilitate tumor expansion and metastasis. This review summarizes the role of TAM-related signaling pathways in driving immune evasion and ICI resistance in HCC, with particular emphasis on the contribution of TAM surface receptors and chemokines in immune suppression. Additionally, the review highlights emerging insights into TAM metabolic reprogramming and transcriptional regulation, which have been closely linked to ICI resistance. Furthermore, we explore promising therapeutic strategies targeting TAMs and their associated signaling pathways to enhance ICI efficacy in HCC. Integrating these novel approaches could potentially overcome TAM-driven immune evasion and ICI resistance, boosting the efficacy of immunotherapy and improving patient prognosis in HCC.

## Introduction

Hepatocellular carcinoma (HCC), responsible for 75–85% of liver cancer cases, poses a significant challenge to global healthcare [1]. Both its incidence and mortality rates of HCC are rising worldwide, particularly in Asia and sub-Saharan Africa, due to the high prevalence of hepatitis B (HBV) and hepatitis C (HCV) infections [2]. Additionally, non-alcoholic fatty liver disease (NAFLD) and alcoholic liver disease are also major contributing factors to HCC [3, 4]. Each year, approximately 906,000 new cases of HCC are diagnosed globally, with more than 830,000 deaths, making it a leading cause of cancer-related death [5]. HCC’s high mortality rate is largely attributed to the difficulty of early detection, as most patients are diagnosed in advanced stages with limited treatment options. Although surgical intervention, liver transplantation, transarterial chemoembolization (TACE), and targeted therapies can help prolong patient survival, the overall prognosis remains poor.

Immunotherapy has emerged as a promising therapeutic strategy for HCC, especially in advanced or unresectable stages where conventional therapies have limited efficacy [6]. Central to this approach is immune checkpoint inhibitors (ICIs), notably PD-1 and PD-L1 inhibitors, which have attracted significant attention for their role in reinvigorating T cell responses and restoring antitumor immunity [7]. Clinical trials have demonstrated the efficacy of ICIs, with agents such as nivolumab and pembrolizumab securing approval for advanced HCC treatment [8]. The integration of these immunotherapeutics with existing modalities, including targeted therapies and chemotherapy, represents a promising avenue for enhancing treatment outcomes and broadening therapeutic possibilities for HCC patients [9].

Despite the significant advances in ICIs for HCC, many patients continue to experience primary or acquired resistance [10], highlighting the complex challenge of immune evasion in this malignancy. Recent research has focused on elucidating the mechanisms driving this resistance, with tumor-associated macrophages (TAMs) emerging as a key player [11]. Particularly, M2-polarized TAMs exert immunosuppressive effects in the HCC tumor microenvironment (TME) by secreting anti-inflammatory cytokines, which dampen the antitumor activity of effector T cells, thereby diminishing the efficacy of ICIs [12, 13]. TAMs also promote angiogenesis and epithelial-mesenchymal transition (EMT), processes that enhance tumor invasiveness and further reduce the effectiveness of ICIs [14–16].

To address these barriers, a range of therapeutic strategies is under investigation. Targeting colony-stimulating factor 1 receptor (CSF-1R) has shown promise in reducing TAM accumulation and survival, inhibiting their polarization toward the M2 phenotype, and alleviating their immunosuppressive impact on the TME [17, 18]. Additionally, reprogramming TAMs to shift towards the pro-inflammatory, anti-tumor M1 phenotype presents a compelling strategy [19]. Combination therapies, particularly with anti-angiogenic agents, can inhibit the VEGF signaling pathway, reducing TAM recruitment and improving the TME to enhance immunotherapy responses [20, 21]. Meanwhile, novel approaches such as CXCR4/CXCL12 axis inhibitors and nanoparticle-based drug delivery systems are being explored to selectively target TAMs and improve ICI efficacy in HCC [22–24]. This review will examine the role of TAMs in mediating ICI resistance in HCC, with a focus on the mechanisms of primary and acquired resistance, and will explore emerging therapeutic strategies aimed at overcoming these challenges to improve HCC outcomes.

## ICIs in HCC therapy

ICIs have revolutionized cancer therapy, including for HCC, by targeting immune checkpoints that regulate T cell responses and preventing tumors from evading immune surveillance. In HCC, several ICIs targeting distinct immune checkpoint pathways have shown promising clinical results (Table 1).

### PD-1/PD-L1 inhibition

The PD-1/PD-L1 axis is central in HCC immune evasion [25]. PD-1, expressed on T cells, interacts with PD-L1 on tumor cells, leading to T cell exhaustion. Inhibitors such as nivolumab and pembrolizumab (anti-PD-1), and atezolizumab and durvalumab (anti-PD-L1), have demonstrated clinical efficacy by reactivating T cells and restoring antitumor immunity [26–29]. These agents function by binding to the PD-1 receptor on T cells or PD-L1 on tumor cells, effectively blocking the PD-1/PD-L1 interaction. This disruption alleviates immune suppression on T cells, thereby reactivating their anti-tumor response. Moreover, combination therapies of anti-PD-1/PD-L1 with anti-angiogenic agents (e.g., bevacizumab) have shown superior efficacy compared to traditional treatments like sorafenib, representing a major advancement in therapeutic strategies for HCC [30].

### CTLA-4 inhibition

CTLA-4 is another immune checkpoint that downregulates T cell activation [31]. Ipilimumab, a monoclonal antibody targeting CTLA-4, works by blocking the interaction between CTLA-4 and its ligands, B7-1 and B7-2, on antigen-presenting cells (APCs) [32]. This blockade releases the inhibitory signal on T cells, enhancing their activation and proliferation. Ipilimumab promotes T cell priming in lymph nodes, allowing for a stronger and more sustained anti-tumor immune response. When combined with PD-1 inhibitors, such as nivolumab, this synergistic approach further amplifies T cell activation, improving tumor-specific immune responses [33]. Cadonilimab, a bispecific antibody targeting both PD-1 and CTLA-4, enhances T cell mediated anti-tumor immunity, offering a potent anti-tumor effect. This promising therapy has demonstrated encouraging efficacy and a manageable safety profile in advanced HCC [34–36].

### LAG-3 and TIM-3 inhibition

Lymphocyte activation gene-3 (LAG-3) and T cell immunoglobulin and mucin-domain containing-3 (TIM-3) are co-inhibitory receptors that contribute to immune suppression in the TME [37]. LAG-3, expressed on activated T cells, dampens T cell responses by binding to its ligand MHC class II molecules on APCs, leading to T cell exhaustion [38]. Similarly, TIM-3, primarily found on exhausted T cells, interacts with its ligands such as galectin-9, triggering downstream signaling pathways that inhibit T cell activation and cytokine production [39]. Targeting these receptors with monoclonal antibodies is an emerging strategy to further restore T cell function. Clinical trials have indicated that LAG-3 blockade can enhance the effects of PD-1 inhibitors [40], while TIM-3 inhibition shows potential in overcoming resistance to PD-1/PD-L1 therapies [41].

### TIGIT inhibition

T-cell immunoreceptor with Ig and ITIM domains (TIGIT) is an immune checkpoint receptor that dampens T cell responses [42]. TIGIT is expressed on activated T cells and natural killer (NK) cells, where it interacts with its ligands CD155 on tumor cells and APCs [43]. This binding suppresses T cell activation and effector function, contributing to tumor immune evasion. Anti-TIGIT therapies are being investigated as a means to improve the effectiveness of PD-1/PD-L1 inhibitors by blocking this immunosuppressive pathway, further promoting T cell activity [44].

### VISTA inhibition

V-domain Ig suppressor of T cell activation (VISTA), also referred to as PD-1 homolog or Dies1, functions as a ligand on APCs to inhibit T cell activation, thereby promoting tumor progression in cancers liking melanoma, breast, lung, liver, and pancreatic cancer [45, 46]. By modulating immune suppression within the TME, VISTA plays a key role in immune evasion. A Phase I trial (NCT02812875) has shown promising efficacy of CA-170, an inhibitor targeting both VISTA and PD-1/PD-L1 in advanced solid tumors [47]. However, the clinical implications of VISTA inhibition, whether alone or in combination with other therapies, remain under investigation.

### B7-H3 inhibition

B7-H3 (CD276) is overexpressed in various solid cancers, including HCC [48], and correlates with increased recurrence risk and poor prognosis. Targeting B7-H3 with enoblituzumab, a monoclonal antibody, enhances NK cell-mediated antibody-dependent cell-mediated cytotoxicity (ADCC) by promoting IFN-γ expression on NK cells and upregulating PD-L1, strengthening immune responses. When combined with anti-PD-1 therapy, enoblituzumab further amplifies IFN-γ secretion and enhances its production in both NK and CD8^+^ T cells [49], supporting the rationale for their combined use to improve anti-tumor immunity.

**Table 1:** ICIs in HCC therapy

Target	Mechanism of Action	Drug Name	Indications	Approval Status	Ref.
PD-1	Blocks PD-1 receptor to restore T cell function	Nivolumab	Advanced HCC after sorafenib failure	FDA approved (2017)	[26]
		Pembrolizumab	Advanced HCC as second-line therapy	FDA approved (2018)	[27]
		Tislelizumab	Advanced HCC as first-line therapy	Approved in China (2024)	[50]
		Camrelizumab	Advanced HCC, single or combination therapy	Approved in China (2020)	[51]
		Sintilimab	Combination therapy with IBI305 for advanced HCC as first-line therapy	Approved in China (2021)	[52]
		Toripalimab	Combination therapy with bevacizumab for advanced HCC	In clinical trials	[53]
		Nofazinlimab	Combination therapy with CS1002 for advanced HCC	In clinical trials	[54]
		Dostarlimab	Combination therapy with cobolimab for advanced HCC	In clinical trials	[55]
		Cemiplimab	Neoadjuvant therapy for resectable HCC	In clinical trials	[56]
		Spartalizumab	Combination therapies for progressive or advanced HCC	In clinical trials	[57, 58]
		Penpulimab	Combination therapy with anlotinib for unresectable HCC	In clinical trials	[59]
		Serplulimab	Combination therapy with HLX04 for advanced HCC	In clinical trials	[60]
		Pucotenlimab	Combination therapy with bevacizumab or lenvatinib for advanced HCC	In clinical trials	[61]
PD-L1	Blocks PD-L1 interaction with PD-1 receptor	Atezolizumab	Advanced HCC with bevacizumab	FDA approved (2020)	[28]
		Durvalumab	Advanced HCC, combination therapy	In clinical trials	[29]
		Avelumab	Advanced HCC in clinical trials	In clinical trials	[62]
		Sugemalimab	Advanced HCC in clinical trials	In clinical trials	[63]
		Envafolimab	Advanced HCC in clinical trials	In clinical trials	[64–66]
		Adebelimumab	Advanced HCC in clinical trials	In clinical trials	[67]
		TQB2450	Advanced HCC in clinical trials	In clinical trials	[68]
CTLA-4	Blocks CTLA-4 to enhance T cell activation	Ipilimumab	Advanced HCC with nivolumab combination	FDA approved (2020, combination)	[33]
		Tremelimumab	Advanced HCC with durvalumab combination	In clinical trials	[29, 69]
PD-1/CTLA-4	PD-1/CTLA-4 bispecific antibody enhancing T cell mediated anti-tumor immunity	Cadonilimab	Combination therapy with lenvatinib for advanced HCC	In clinical trials	[34–36]
LAG-3	Blocks LAG-3 pathway to restore T cell activation	Relatlimab	Emerging checkpoint target in clinical trials	In clinical trials	[70]
TIM-3	Blocks TIM-3 interaction to prevent T cell exhaustion	Sabatolimab	Emerging checkpoint target in clinical trials	In clinical trials	[41]
TIGIT	Blocks TIGIT interaction with CD155 to enhance T cell function	Tiragolumab	Emerging checkpoint target in clinical trials	In clinical trials	[71]
VISTA	Blocks VISTA to restore T cell function	CA-170	Emerging checkpoint target in clinical trials	In clinical trials	[47]
B7-H3	Targets B7-H3 to enhance NK cell-mediated ADCC	Enoblituzumab	Emerging checkpoint target in clinical trials	In clinical trials	[49]

## Predictive biomarkers for immunotherapy efficacy

HCC is a highly heterogeneous malignancy. Although tissue biopsy remains the gold standard for diagnosis, it is hard to fully capture the tumor’s genetic landscape and impractical for repeated use, especially in monitoring treatment response during immunotherapy. Liquid biopsy, due to its non-invasive nature, has thus gained increasing attention as a promising alternative for monitoring treatment responses in ICIs therapy. This approach, including the analysis of circulating tumor DNA (ctDNA), circulating tumor cells (CTCs), lymphocyte subpopulations, exosomes, and metabolites, offers a dynamic method for real-time tracking of tumor evolution [72–75].

Both ctDNA and CTCs have been shown to accurately reflect the tumor genome [76]. ctDNA is quantifiable across all stages of HCC [77], enabling detection of key driver mutations that are crucial for tumor progression. Moreover, elevated CTC loads are associated with a higher probability of tumor recurrence [78]. Thus, liquid biopsy provides an invaluable tool to dynamically monitor genomic changes throughout treatment, offering insights into therapeutic efficacy and disease progression.

Recent studies have revealed the utility of ctDNA and CTCs in assessing ICI treatment responses. In lung and urothelial cancers, changes in somatic mutation allele frequencies within ctDNA correlate with ICI treatment duration, activity, and overall outcomes [79]. Similarly, CTC counts, particularly when coupled with PD-L1 expression, have indicated potential in predicting urothelial cancer progression in patients undergoing ICI treatment [80]. Moreover, ctDNA levels can serve as early markers of therapeutic efficacy, with significant declines in ctDNA correlating with prolonged survival in lung cancer patients treated with ICIs [81]. These findings align with recent prospective cohort studies on HCC, where elevated cfDNA and ctDNA levels have been linked to poorer overall survival (OS), lower objective response rate (ORR), and shorter progression-free survival (PFS) in advanced HCC patients treated with immunotherapy [82, 83]. ctDNA analysis also offers a valuable marker for assessing disease progression during lenvatinib treatment in HCC [84].

In addition, the genomic profile of HCC can be further elucidated by sequencing ctDNA specific mutations. For example, CTNNB1 mutations, known to activate the Wnt/β-catenin pathway, have been identified as significant predictors of response to immunotherapy [85, 86]. These mutations are frequently detected in ctDNA, which, when combined with tumor tissue analysis, improves detection rates [87]. Furthermore, circulating exosomes containing PD-L1 mRNA and protein levels may offer additional predictive value for response to anti-PD-1/PD-L1 therapies [88, 89].

## ICI resistance in HCC

ICIs, particularly those targeting the PD-1/PD-L1 and CTLA-4 pathways, have significantly reshaped cancer immunotherapy and have demonstrated transformative effects across various malignancies. However, despite the success of ICIs in other cancers, their clinical benefits in HCC remain limited, with ORR typically ranging from 15 to 23% [90, 91]. The limited effectiveness of ICIs in HCC raises critical questions about the mechanisms driving both primary and acquired resistance to these therapies [91, 92]. Primary resistance refers to the failure of ICIs to induce a meaningful clinical response at the onset of treatment. This form of resistance is commonly associated with a non-inflamed TME, characterized by insufficient T cell infiltration, limited tumor antigen presentation, and the dominant immunosuppressive pathways. Additionally, intrinsic tumor factors such as low tumor mutational burden (TMB) can also contribute to immune exclusion and lack of response [93–95]. In contrast, acquired resistance arises when patients initially respond to ICIs but subsequently experience disease progression. This can result from dynamic adaptations within the tumor or immune system, including the upregulation of alternative immune checkpoints, loss of neoantigens, or clonal evolution under immune pressure [96–98]. Understanding the distinct and overlapping mechanisms of primary and acquired resistance is essential for developing effective combination strategies to overcome immune evasion and improve outcomes in HCC.

**Fig. 1:**
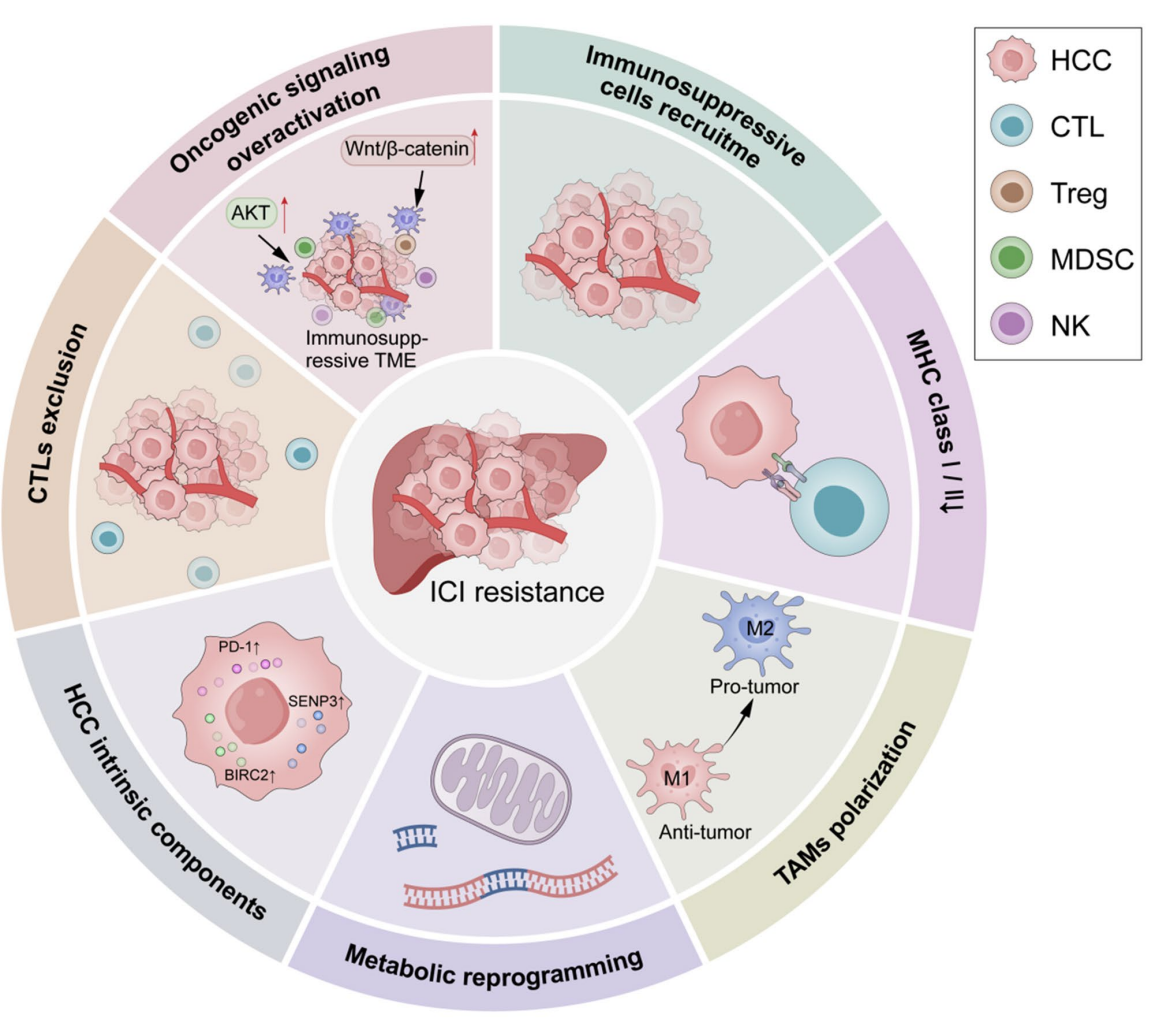
Immunosuppressive TME in HCC and ICI resistance. ICI resistance in HCC arises from a multifactorial immunosuppressive landscape. The recruitment of Tregs and MDSCs into the TME suppresses CTL activity. Simultaneously, reduced MHC I/II expression on tumor cells impairs antigen presentation, compromising immune recognition. CTL exclusion is further driven by TME barriers and vascular remodeling. TAMs, predominantly polarized to the M2 phenotype, secrete immunosuppressive cytokines and promote immune-suppressive cell infiltration. Additionally, metabolic reprogramming driven by hypoxia and lactate accumulation further suppresses T cell activity. The overactivation of oncogenic pathways promotes immune escape by hindering both T cell infiltration and function. Collectively, these mechanisms converge to impair ICI efficacy

One of the key obstacles to ICI effectiveness in HCC lies in the unique immunosuppressive characteristics of its TME [99]. HCC is often described as an “immune-cold” tumor, characterized by a scarcity of cytotoxic T lymphocytes (CTLs) within the tumor lesions [100]. Instead, HCC lesions are frequently populated by immunosuppressive cell populations, including regulatory T cells (Tregs) and myeloid-derived suppressor cells (MDSCs) [101–103], which suppress the immune response and hinder T cell-mediated tumor killing. Elevated levels of inhibitory cytokines within the TME further dampen the function of immune cells, compounding the challenges of effective ICI treatment [99]. The TME in HCC also features reduced expression of MHC class I (MHC-I) molecules on tumor cells, which impairs antigen presentation and reduces the capacity of NK or T cells to recognize and eliminate tumor cells [104–106]. This inability to properly present tumor-associated antigens results in an insufficient antitumor response, further contributing to resistance to ICIs [106]. In addition, key oncogenic signaling pathways, such as Wnt/β-catenin and AKT [11, 107], play a significant role in shaping the immunosuppressive environment. These pathways hinder T cell infiltration into tumors and promote the expansion of immunosuppressive subsets, further fostering resistance to immunotherapy. The metabolic reprogramming within the TME also significantly contributes to ICI resistance. Hypoxic conditions, along with the accumulation of lactate, create a metabolically hostile environment that severely impairs T cell function [108, 109]. These metabolic alterations compromise the ability of T cells to mount a robust immune response. Beyond the above barriers, tumor-intrinsic components also critically orchestrate immune resistance in HCC. BIRC2 drives the ubiquitin-mediated degradation of NF-κB-inducing kinase (NIK), attenuating non-canonical NF-κB signaling and downregulating MHC-I expression to evade CTL recognition [110]. SENP3 stabilizes RACK1 and enhances eIF4E-dependent translation of oncogenes and CCL20, facilitating TAMs recruitment and diminishing CTLs infiltration [111]. Remarkably, PD-1 expression in HCC cell extends beyond its classical role, directly activating mTOR signaling via eIF4E and S6 phosphorylation to support tumor growth independent of adaptive immunity [112] (Fig. 1).

Against this backdrop, TAMs have emerged as crucial mediators of ICI resistance in HCC. These macrophages primarily exhibit an M2-like phenotype, which is closely associated with immune evasion and tumor progression [113–115]. TAMs play a central role in shaping the immunosuppressive TME by secreting a variety of pro-tumor cytokines and and recruiting immunosuppressive populations, such as Tregs, which further suppress anti-tumor immune responses and facilitate tumor metastasis [116]. Multiple molecular pathways regulated by TAMs have been identified as critical drivers of ICI resistance. Galectin-1 (Gal1), secreted by tumor cells, has been shown to exacerbate TAM-driven immune suppression. Through activation of the PI3K/AKT/NF-κB pathway, Gal1 stimulates TAMs to secrete CCL20, which subsequently recruits CCR6^+^Foxp3^+^ Tregs and impairs CD8^+^ T cell function. Targeting Gal1 leads to a reduction in CCL20 secretion, thus inhibiting Treg recruitment and enhancing the response to anti-PD-1 therapy [117]. Leukotriene A4 hydrolase (LTA4H), an inflammatory mediator, is significantly downregulated in HCC. The loss of LTA4H impairs JNK activation and promotes the polarization of CD206^+^ macrophages via upregulation of LTBP1, which activates TGF-β signaling, contributing to resistance to immunotherapy [118]. Moreover, TAMs undergo metabolic reprogramming, contributing to ICI resistance. CircPETH, a circular RNA transferred from TAMs to HCC cells via extracellular vesicles, promotes glycolysis and metastasis. The CircPETH-147aa variant enhances PKM2-mediated ALDOA-S36 phosphorylation, inhibiting anti-HCC immune responses. This process occurs by stabilizing SLC43A2 mRNA through a HuR-dependent mechanism, resulting in the depletion of methionine and leucine in cytotoxic CD8^+^ T cells, thereby impairing their functionality [119]. Macrophage-specific phospholipase A2 Group VII (PLA2G7) expression correlates with poorer prognosis and immunotherapy resistance. Inhibition of PLA2G7 improves anti-PD-1 efficacy, offering a novel approach to enhance ICI responses [120]. Additionally, macrophage-coated tumor clusters (MCTCs) contribute to ICI resistance by trapping immunocompetent cells and excluding cytotoxic T cells. Tumor-derived macrophage-associated lectin M2BP induces MCTC formation, and blocking M2BP enhances T cell infiltration and ICI therapy efficacy [121]. These mechanisms collectively reinforce an immunosuppressive landscape that hampers T cell function, diminishes the effectiveness of ICIs, and ultimately promotes tumor growth and progression. Investigating strategies to overcome the immunosuppressive barriers posed by TAMs may hold the key to improving the clinical efficacy of ICIs in HCC.

## Polarization of TAMs in HCC: signaling pathways and functional implications

TAMs are pivotal in shaping the liver cancer microenvironment, where they can either bolster anti-tumor immunity or, more commonly, promote tumor progression through immunosuppressive mechanisms [122–124]. TAMs arise from two primary sources: bone marrow-derived monocytes and liver-resident Kupffer cells [125, 126]. In response to inflammatory signals or tumor-derived cues, circulating monocytes are recruited to the TME by chemokines such as CCL2 and CCL5, where they differentiate into TAMs [127]. Kupffer cells, which typically maintain liver homeostasis by clearing pathogens and apoptotic cells, can be reprogrammed under tumor conditions, adopting pro-tumoral functions [128].

**Fig. 2:**
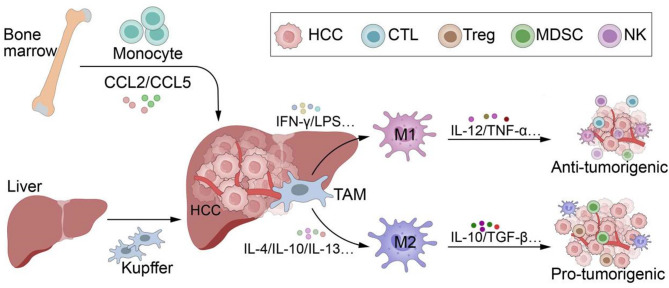
Sources and polarization of TAMs in HCC. TAMs play a critical role in shaping HCC TME. TAMs originate from two main sources: bone marrow-derived monocytes and resident Kupffer cells in the liver. Monocytes are recruited to the tumor site by chemokines, where they differentiate into TAMs, while Kupffer cells can be reprogrammed in the tumor context to acquire pro-tumor functions. TAMs exhibit plasticity and can polarize into pro-inflammatory M1 or immunosuppressive M2 phenotypes. M1 macrophages promote anti-tumor immunity by secreting pro-inflammatory cytokines and inducing tumor cell death. In contrast, M2 macrophages suppress immune responses, and support tumor growth, angiogenesis, and metastasis

TAMs exhibit functional plasticity, existing along a polarization spectrum between pro-inflammatory M1 and immunosuppressive M2 phenotypes [129]. M1 macrophages, activated by signals such as IFN-γ and LPS, play a tumor-suppressive role by producing pro-inflammatory cytokines like IL-12 and TNF-α, promoting effector T cell responses, inducing tumor cell apoptosis, and inhibiting angiogenesis [130–134]. Conversely, M2 macrophages, polarized by cytokines such as IL-4, IL-10, and IL-13, contribute to a pro-tumoral microenvironment by secreting immunosuppressive factors like IL-10 and TGF-β, dampening anti-tumor immune responses, promoting tissue remodeling, and facilitating tumor growth, angiogenesis, and metastasis [134–136] (Fig. 2). In the TME of liver cancer, TAM polarization is regulated by a variety of signaling pathways, which drive the transformation of TAMs toward the pro-tumoral M2 phenotype.

### NF-κB signaling pathway

Several studies investigate the pivotal role of NF-κB in the polarization of TAMs towards a pro-tumor M2 phenotype in HCC. One study identifies a crucial HMGB1/TLR2/NOX2/autophagy signaling axis involved in this process. Specifically, HMGB1 released by HCC cells activates TLR2, leading to the activation of NOX2 and subsequent ROS production. This ROS generation suppresses NF-κB p65 activity, favoring M2 macrophage polarization and fostering tumor growth [137]. Another study found that elevated levels of Drp1 are associated with an increased presence of CD163-positive M2-like TAMs. Mitochondrial fission induces mitochondrial DNA (mtDNA) stress, releasing mtDNA into the cytosol and triggering CCL2 secretion through the TLR9 mediated NF-κB pathway, promoting TAM recruitment. Targeting this pathway with DNase I or TLR9 antagonists effectively reduces TAM infiltration and slows HCC progression [138]. NF-κB p65 also regulates macrophage polarization through its interaction with the transcription factor Zhx2 in the TME. In HCC, Zhx2 deletion promotes pro-tumor TAM phenotypes, and lower Zhx2 levels in TAMs are linked to poor survival in patients. Mechanistically, Zhx2 binds to NF-κB p65 and the Irf1 promoter, driving Irf1 transcription, which is essential for maintaining macrophage polarization [139]. Additionally, the tumor suppressor NDRG2 influences TAM polarization through NF-κB signaling. In Ndrg2-deficient mice, increased M1-like TAMs are associated with suppressed liver tumor growth. Inhibition of IκBα phosphorylation diminishes M1 marker expression, emphasizing NF-κB’s pivotal role in the tumor-suppressive functions of NDRG2-deficient macrophages. Meanwhile, TLR2-related ligands from HCC cells sustain the M2 phenotype by promoting the ubiquitination and degradation of NF-κB RELA/p65 [140]. These findings illuminate the complex regulatory network surrounding NF-κB in TAM polarization, indicating novel therapeutic strategies to modulate TAM behavior and combat HCC progression.

### STAT signaling pathway

The STAT signaling cascade has been recognized as a key modulator of TAM phenotype and activity within the TME of HCC. Recent studies reveal the potential of targeting this pathway to disrupt pro-tumorigenic TAM functions. For instance, enhanced SHP-1 expression in macrophages inhibits tumor progression by preventing M2-like polarization. SHP-1 activates SHP2, counteracting GM-CSF-induced TAM recruitment and attenuating STAT3/6 phosphorylation, thereby reducing the expression of M2 markers such as Arg-1, CD206, and CD163, along with cytokines IL-4 and IL-10 [141]. In advanced HCC, elevated TRIM65 expression correlates with higher tumor grade, stage, and poorer prognosis. Functional experiments demonstrate that TRIM65 knockout diminishes tumor burden, suppresses M2 polarization, and enhances M1-like activation through the JAK1/STAT1 axis [142]. ACKR3 and HDAC2 cooperate to regulate TAM recruitment and polarization in HCC, with elevated levels promoting M2-like migration and immune evasion. Silencing these factors enhances M1 polarization and restores anti-tumor T cell responses. HDAC2 recruits STAT1 to the ACKR3 promoter, driving its transcription and supporting TAM infiltration, while HDAC2 inhibition prevents STAT1 nuclear translocation, reinforcing its role in HCC progression [143]. Additionally, reduced SIRT4 expression correlates with elevated M2 polarization and worse outcomes. Mechanistically, SIRT4 suppression amplifies fatty acid oxidation (FAO)-PPARδ-STAT3 signaling, driving higher MCP-1 production and promoting TAM recruitment [144]. Abnormal expression of B7-H3 in HCC supports heightened M2 marker expression and accumulation of TAMs, resulting in unfavorable survival rates. The mechanism involves B7-H3 activation of STAT3 signaling to support M2 polarization, which is effectively disrupted upon STAT3 inhibition [145]. Together, these findings emphasize the complex interplay between these factors and the broader role of STAT signaling in TAM regulation. Future research should investigate combinatorial targeting approaches to reprogram TAMs and enhance anti-tumor immunity in HCC.

### AKT signaling pathway

The AKT signaling pathway is a crucial regulator of TAM polarization and function, significantly influencing tumor progression in liver cancer. Recent studies emphasize its influence on the immune landscape within the TME. VEGF promotes M2 macrophage polarization by enhancing the expression of markers such as CD163 and CD206 through AKT pathway activation. Inhibition of VEGF has been shown to suppress TAM-driven tumor progression by targeting the AKT/mTOR axis, thereby reducing tumor cell proliferation, migration, and immune evasion. This blockade also reduces the expression of PD-L1 and pro-tumor cytokines [146]. Tumor necrosis factor α-induced protein 8-like 1 (TIPE1), another pivotal factor, regulates TAM alternative activation via the PI3K/AKT pathway by modulating phosphoinositide metabolism (PIP2 and PIP3) [147]. Elevated TIPE1 levels in TAMs correlate with poorer patient survival outcomes. Targeting TIPE1 through the inhibition of TGFβ signaling in macrophages has been shown to reduce its tumor-promoting effects, suggesting potential therapeutic avenues for modulating TAM behavior. Additionally, triggering receptor expressed on myeloid cells 1 (TREM1) further underscores the role of AKT signaling in TAM polarization [148]. Elevated TREM1 expression in HCC, is associated with enhanced tumor progression. Silencing TREM1 induces a shift from M2 to M1 polarization, reducing M2 markers and impairing tumor cell migration and invasion via suppression of the PI3K/AKT/mTOR pathway. This suggests that TREM1, in concert with AKT signaling, plays a significant role in maintaining a pro-tumor TAM phenotype.

### Metabolic signaling pathway

Metabolic pathways are integral to the regulation of TAM polarization in HCC, with emerging evidence suggesting that metabolic reprogramming of TAMs contributes to tumor progression and immune evasion. Recent studies have identified key metabolic signals that drive TAM polarization toward a pro-tumorigenic M2 phenotype, opening new avenues for targeting these metabolic networks to enhance anti-tumor immunity in liver cancer. One key metabolic regulator of TAM polarization in HCC is Pumilio RNA-binding family member 1 (PUM1), which promotes M2 polarization through the cAMP signaling pathway [149]. By driving immune suppression and hindering CD8^+^ T cell-mediated anti-tumor responses, PUM1 plays a pivotal role in fostering a tumor-promoting microenvironment. The absence of PUM1, as demonstrated in knockout mouse models, significantly alters TAM function. Another metabolic regulator central to TAM reprogramming is OXCT1, an enzyme involved in ketolysis [150]. Highly expressed in TAMs, OXCT1 facilitates their polarization towards a pro-tumor phenotype through the succinate-H3K4me3-Arg1 axis. This reprogramming promotes CD8^+^ T cell exhaustion and suppresses anti-tumor immunity. Inhibition of OXCT1, either pharmacologically or through genetic knockout, reduces TAM-mediated immune suppression, enhances T cell cytotoxicity, and slows tumor progression. Iron metabolism is another crucial factor influencing TAM polarization in HCC. Iron deprivation has been shown to drive M2 polarization, with competition for iron between TAMs and HCC cells playing a central role [151]. HCC cells upregulate the transferrin receptor (TFRC), leading to iron depletion in TAMs and activation of HIF-1α, which promotes M2 polarization. Notably, overexpression of TFRC correlates with poor prognosis and increased M2 infiltration in HCC patients. The role of receptor-interacting protein kinase 3 (RIPK3), a key regulator of necroptosis, in lipid metabolism and TAM polarization has also been explored [152]. RIPK3 deficiency in TAMs accelerates tumor progression and enhances M2 polarization, largely by promoting fatty acid metabolism. The absence of RIPK3 reduces reactive oxygen species (ROS) and inhibits the caspase-1-mediated cleavage of PPAR, whicn in turn supports M2 polarization. Enhancing RIPK3 expression or inhibiting FAO reverses the M2 phenotype. Rencent findings also points to PCSK9, a regulator of cholesterol metabolism, as a key player in TAM polarization in HCC [153]. Decreased expression of PCSK9 within the TME is associated with increased M2 macrophage infiltration, which can be further enhanced by silencing PCSK9. Interestingly, overexpression of PCSK9 inhibits M2 polarization, partially by upregulating OX40L, a co-stimulatory molecule. Inhibition of OX40L reverses this effect, positioning PCSK9 as a crucial modulator of TAM polarization in liver cancer.

### MiRNA mediated signaling pathways

MicroRNAs (miRNAs) are increasingly recognized as critical regulators of TAM polarization in HCC. Specific miRNAs promote M2 polarization of TAMs, facilitating immune evasion, tumor metastasis, and resistance to therapy, thereby driving the progression of HCC. miR-23a-3p, enriched in exosomes derived from M2 macrophages, facilitates metastasis by promoting EMT, enhancing angiogenesis, and increasing vascular permeability [154]. It exerts its effects by targeting key tumor suppressors PTEN and TJP1, thereby fostering a pro-tumor microenvironment. Additionally, these exosomes stimulate HCC cells to release pro-tumor cytokines like GM-CSF, VEGF, and IL-4, which amplify M2 macrophage recruitment and polarization, creating a self-reinforcing cycle that supports tumor progression. Similarly, miR-21-5p, found in exosomes derived from HCC cells, drives TAMs toward the M2 phenotype, upregulating the production of TGF-β and IL-10 [155]. Overexpression of miR-21-5p modulates MAPK signaling via suppression of RhoB, forming a microenvironment conducive to tumor growth. Another miRNA, miR-155, shows high expression in HBV-related HCC [156]. Through the miR-155/SHIP1 axis, it enhances M2 polarization, enabling tumor proliferation, invasion, and immune suppression. Knockdown of miR-155 shifts macrophages to the M1 phenotype, making it an attractive target, especially in HBV-associated HCC. In contrast, the miR-144/miR-451a cluster, identified as a prognostic marker in HCC, influences macrophage polarization by suppressing the secretion of factors such as HGF and MIF from HCC cells [157]. These miRNAs promote M1 polarization of TAMs, enhancing their phagocytic activity and ability to activate CTLs. Interestingly, miR-144/miR-451a operates through a feedback loop with EZH2, a histone methyltransferase, which silences their expression in HCC cells via epigenetic modifications.

### Other signaling pathways

#### Hedgehog signaling pathway

Hedgehog (Hh) signaling in TAMs contributes to tumor growth by driving M2 polarization and suppressing antitumor immunity. Tumor-derived SHH activates the Hh-Gli1-Klf4 signaling axis in TAMs, leading to reduced production of CXCL9 and CXCL10, chemokines essential for CD8^+^ T cell infiltration into the TME [158]. This creates an immunosuppressive niche that enhances tumor progression. M2-polarized TAMs further secrete immunosuppressive cytokines such as IL-10 and TGF-β while expressing immune checkpoint ligands, exacerbating CD8^+^ T cell dysfunction. Importantly, inhibiting Hh signaling reverses M2 polarization, restores T cell infiltration, and synergizes with immune checkpoint blockade to suppress tumor growth.

#### PTEN signaling pathway

One study explores the role of growth arrest-specific 5 (GAS5) in the polarization of TAMs in HCC, highlighting its relationship with the PTEN signaling pathway. GAS5 is found to be upregulated in M1 macrophages and downregulated in M2 macrophages and TAMs [159]. Overexpression of GAS5 promotes M1-like polarization and enhances PTEN expression in TAMs, effectively inhibiting M2 polarization. Conversely, the supernatant from PTEN silenced TAMs increases the proliferation and invasion of SMCC-7721 cells, diminishing the inhibitory effects of GAS5, suggesting GAS5 modulating TAM polarization through the PTEN pathway.

#### β-adrenergic receptor signaling pathway

A recent study reported that chronic stress promotes M1 to M2 polarization of TAMs, mediated by β-adrenergic signaling pathway, enhancing immunosuppression and tumor growth [160]. Stress-induced increases in norepinephrine and epinephrine levels mobilize CCR2^+^ monocytes from bone marrow to tumor sites, contributing to TAM infiltration. Additionally, the CCL2/CCR2 axis plays a vital role in recruiting macrophages and driving M2 polarization within the TME. Propranolol effectively blocks stress-induced increases in CCR2^+^ monocytes and CCL2 levels, disrupting TAM-mediated immunosuppression.

#### Calcium-binding protein family

Calcium-binding proteins have emerged as central regulators of the TME, directing macrophage polarization to facilitate immune evasion and tumor progression in liver cancer. Elevated expression of S100A9 in TAMs drives their polarization toward the M2 phenotype, thereby enhancing liver cancer cell proliferation, migration, invasion, and EMT. Suppression of S100A9 disrupts this process, reducing M2 polarization and impairing tumor progression [161]. Complementary single-cell RNA sequencing (scRNA-seq) of HCC samples from nine HBV-associated patients revealed a stemness-related malignant subclone expressing CD24, CD47, and ICAM1, associated with poorer survival. Functional validation identified S100A11 as a critical mediator of tumor initiation and stemness maintenance. Moreover, this subclone establishes bi-directional communication with TAMs, promoting M2-like polarization and sustaining cancer stem cell markers [162].

#### Transcriptional regulators

One study utilizing single-cell transcriptomics identified the transcription factor PRDM1 as a pivotal regulator that drives TAM polarization towards the M2 phenotype [163]. This polarization is significant in establishing an immunosuppressive environment that facilitates tumor growth and progression. Experimental validation of PRDM1’s role underscores its potential as a target for therapeutic interventions aimed at altering TAM behavior in HCC. The forkhead box O1 (FoxO1) transcription factor has been implicated in the regulation of TAMs as well [164]. TAMs exhibiting lower levels of FoxO1 were associated with increased M2 polarization, a phenomenon driven by hypoxic conditions within the TME. FoxO1 positively regulates MHC-II gene expression, and its absence in TAMs leads to decreased MHC-II levels, further promoting tumor growth. Targeting FoxO1 could therefore represent a novel approach to enhance anti-tumor immune responses by reprogramming TAMs towards a more pro-inflammatory phenotype.

#### CSF-1R

Colony-stimulating factor-1 (CSF-1) and its receptor (CSF-1R) are crucial for macrophage differentiation and infiltration in HCC. PLX3397, a CSF-1R inhibitor, promotes the polarization of TAMs from an M2 phenotype to an M1 phenotype, delaying tumor growth and improving survival in hepatoma models [165]. The transition is mediated by inhibiting CSF-1R signaling, which reduces the protumorigenic effects of TAMs without depleting them. PLX3397 treatment enhances antitumor immunity by increasing antigen-presenting macrophages and CD8^+^ T cells while reducing MDSCs and CD4^+^ T cells in the TME. Additionally, elevated levels of CSF2, IL3, and IFNγ suggest adaptive changes in the TME.

**Fig. 3:**
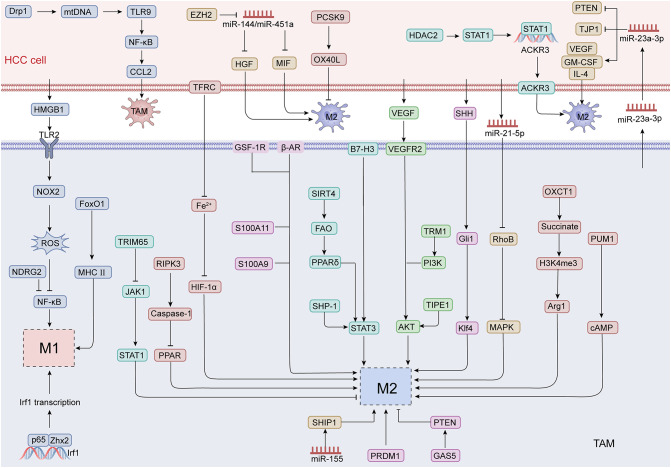
Signaling pathways driving TAMs polarization in HCC. In the TME of HCC, multiple signaling pathways drive the polarization of TAMs towards M2 phenotype, further intensifying immune suppression and promoting tumor progression. These pathways alter TAM function and contribute to the creation of a pro-tumor microenvironment that facilitates immune evasion and enhances tumor growth

In summary, these studies collectively highlight the multifaceted signaling pathways influencing TAM polarization in HCC (Fig. 3). From the regulatory roles of PRDM1, S100A proteins, and PCSK9 to the implications of FoxO1 and CSF-1R, these results pave the way for novel therapeutic approaches aimed at modulating TAMs and enhancing immune responses in liver cancer. Further exploration of these pathways could lead to more effective treatments that improve patient outcomes in HCC.

## TAM-mediated mechanisms of resistance to ICIs

### The crosstalk between TAMs and other immune cells in ICI resistance

TAMs serve as pivotal regulators in shaping the TME through interactions with various immune cells and stromal components, including tumor-associated neutrophils (TANs), NK cells, T cells and cancer-associated fibroblasts (CAFs). These dynamic cellular interactions contribute to the establishment of an immunosuppressive microenvironment, a critical mechanism underlying resistance to ICIs.

In HCC and other solid tumors, tumor-derived signals induce neutrophils to adopt an immunosuppressive phenotype, transforming them into TANs. These TANs accelerate CD8⁺ T cell exhaustion and recruit TAMs and Tregs, further enhancing immune escape [166–169]. TAMs also diminish NK cell cytotoxicity through the secretion of various cytokines, including ARG1, IDO, IL-10, and TGF-β, while upregulating immune checkpoint ligands and immunosuppressive factors that suppress T cell effector functions [170–172]. Crucially, the crosstalk between TAMs and CAFs exacerbates NK cell suppression, therefore weakening anti-tumor immunity and fostering immune tolerance within the TME [173].

Among these cellular interactions, the TAM-CAF crosstalk is considered to be one of the most significant axes driving immune evasion and ICI resistance. CAFs secrete cytokines such as IL-6, IL-33, and IL-10, along with chemokines like CXCL12, CXCL16, and CCL2, which facilitate the polarization of monocytes into M2-like TAMs, thereby promoting an immunosuppressive phenotype [174]. Moreover, CAF-derived IL-6 induces myeloid immunosuppression by activating STAT3 signal. Inhibiting STAT3 activation or the IL-6 pathway has been shown to significantly reduce immune suppression in HCC [175]. In addition to IL-6, IL-11 secreted by CAFs also activates STAT3, facilitating the differentiation of TAMs into a tumor-supportive M2-like phenotype [176]. Although CAFs promote the polarization of M2-like TAMs, TAMs reciprocally influence the differentiation and activation of fibroblasts, directing them towards a more tumor-promoting phenotype [177]. This bidirectional regulatory axis between TAMs and CAFs has been established as a key mechanism in modulating immune status and driving resistance to ICIs.

Recent advances in single-cell transcriptomics have unveiled the functional crosstalk between TAMs, CAFs, and T cells. Bioinformatics analysis of 43 tumor samples from 14 HCC patients and 14 adjacent control tissues revealed that SPP1⁺ APOC1⁺ TAMs highly co-localize with CAFs, and SPP1 binds to ITGF1 secreted by CAFs, collectively remodeling the tumor immune microenvironment. Furthermore, FAP⁺ CAFs engage with naïve T cells via the CXCL12-CXCR4 axis, potentially contributing to ICI resistance [178].

### TAM receptors in ICI resistance

**Fig. 4:**
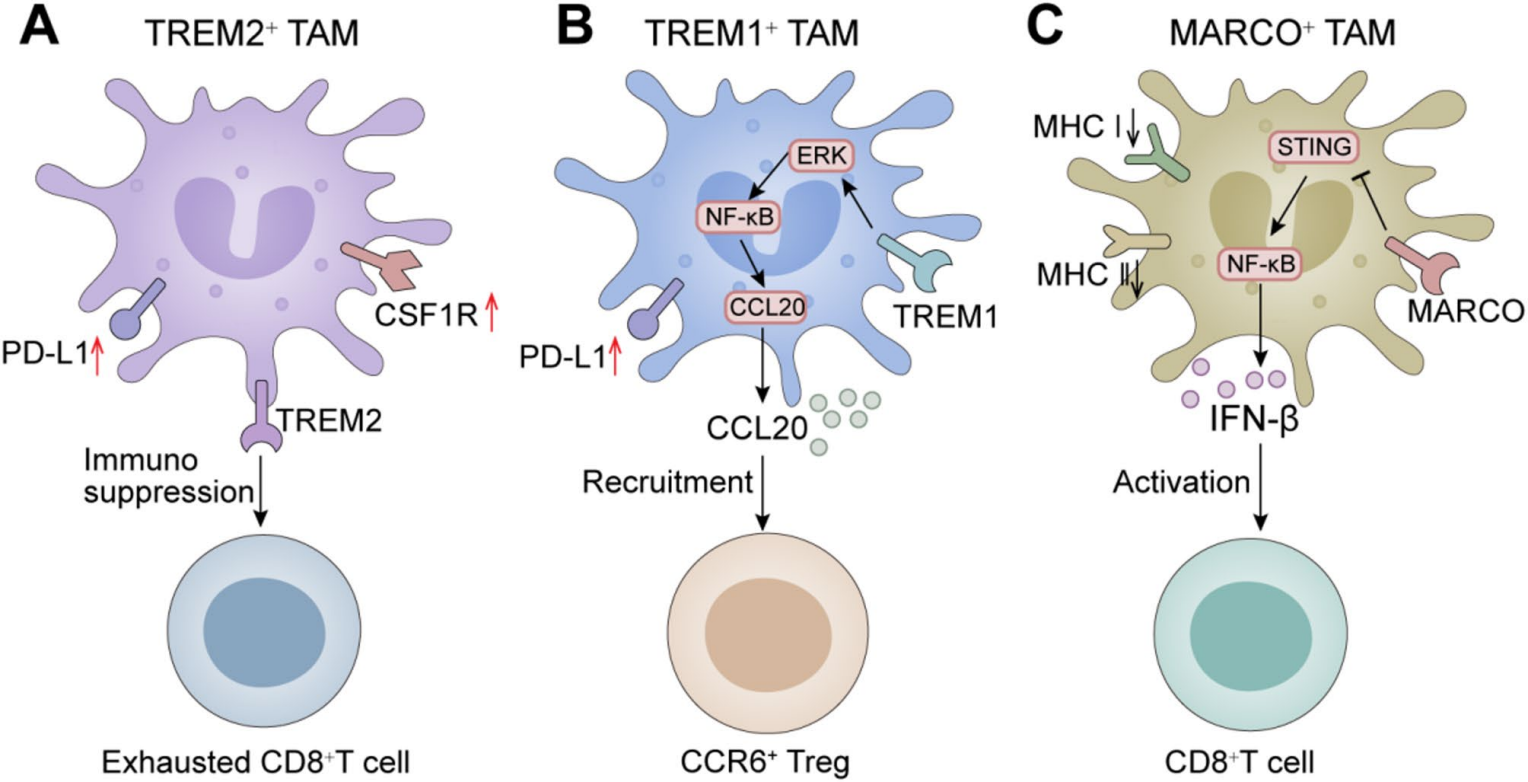
TAMs receptors in ICI resistance. (**A**) TREM2^+^ TAMs in the TME express immunosuppressive signals, leading to CD8^+^T cell exhaustion and reduced PD-1 therapy efficacy. Targeting TREM2^+^ TAMs with anti-Csf1r antibodies enhances PD-1 response. (**B**) In advanced HCC, TREM1^+^ TAMs, induced by hypoxia, recruit CCR6^+^Tregs and suppress CD8^+^ T cell activity, contributing to resistance to PD-L1 blockade. Blocking TREM1 improves PD-L1 therapy. (**C**) MARCO^+^ TAMs inhibit IFN-β secretion, impair antigen presentation, and promote immune suppression, contributing to ICI resistance. Combining MARCO inhibition with PD-L1 blockade suppresses liver cancer growth

Specific surface receptors on macrophages are essential for regulating their function, thereby influencing resistance to ICIs. It has been demonstrated that TREM2^+^ macrophages in the TME expressed immunosuppressive signals, promoting T cell exhaustion and senescence, forming an “immune barrier.” In contrast, TCR^+^ macrophages exhibited tumor-killing abilities. Targeting TREM2^+^ macrophages with anti-Csf1r monoclonal antibodies enhanced PD-1 therapy efficacy. TREM2 knockdown in TAMs reprogrammed them into an immune-stimulating state, improving PD-1 inhibitor responses [17] (Fig. 4A). Additionally, hypoxia-induced TREM-1 expression in TAMs worsened immunosuppression in advanced HCC. TREM-1^+^ TAMs impaired CD8^+^ T cell cytotoxicity, induced T cell apoptosis, and upregulated PD-L1 expression independent of the PD-L1/PD-1 axis. These macrophages also recruited Tregs via CCL20 through the ERK/NF-κB pathway, contributing to resistance against anti-PD-L1 therapy. Blocking TREM-1 reduced Treg recruitment, suppressed tumor progression, and enhanced PD-L1 blockade efficacy [172] (Fig. 4B). MARCO is another surface receptor expressed on macrophages. MARCO^+^ TAMs drive immune suppression by impairing IFN-β secretion, antigen presentation, and CD8^+^ T cell infiltration. They also inhibit the STING-IFN-β pathway, promoting immune dysfunction and ICI resistance. MARCO targeting combined with PD-L1 blockade reduces liver cancer growth in animal models [179] (Fig. 4C).

### Chemokine-mediated resistance to ICIs

Key chemokines and their receptors play a crucial role in driving TAM-mediated immune suppression, which contributes significantly to resistance against ICIs. In HCC models, CX3C motif chemokine receptor 1 (CX3CR1)^+^ TAMs induce T cell exhaustion via IL-27 secretion. Tumor-derived prostaglandin E2 (PGE2) triggers TAMs to adopt a CX3CR1^+^ phenotype, exacerbating immune evasion and impairing ICI efficacy [180]. Similarly, CCL2 and CCL5 are essential for TAM recruitment and polarization. In SLAMF7-deficient HCC cells, CCL2 expression is elevated via the MAPK/ATF2 pathway, enhancing the immunosuppressive M2 phenotype and impairing anti-PD-1 therapy effectiveness. SLAMF7 interacts with SHB and SHIP1 to prevent TRAF6 ubiquitination, inhibiting CCL2 transcription and MAPK activation. Pharmacologically targeting the CCL2/CCR2 axis repolarizes TAMs, improving therapeutic responses in SLAMF7-deficient tumors [13]. A bispecific antibody, BisCCL2/5i, neutralizes both CCL2 and CCL5, reprogramming TAMs from an M2 to an antitumoral M1 phenotype, significantly enhancing PD-1 ligand inhibitor efficacy [127]. In anti-PD-L1 treated HCC patient samples, activation of the renin-angiotensin system promoted tumor progression and resistance. ACE2 overexpression suppressed CCL5 expression and TAM M2 polarization, improving anti-PD-L1 therapy outcomes. Inhibiting CCR5 with maraviroc further enhanced treatment efficacy [181]. Additionally, SYR-Related High-Mobility Group Box 18 (SOX18) induces CXCL12 and PD-L1 expression through TGF-β1 signaling, contributing to ICI resistance by creating a highly immunosuppressiveTME. Targeting CXCR4 or TGFβR1, alongside anti-PD-L1 therapy, reduces TAM and Treg accumulation, suppressing HCC progression [23]. TAMs also contribute to minimal residual disease (MRD) persistence, sustaining stem-like tumor cells and ICI resistance via PD-L1 and TGFβ signaling [12].

### Metabolic reprogramming of TAMs in ICI resistance

**Fig. 5:**
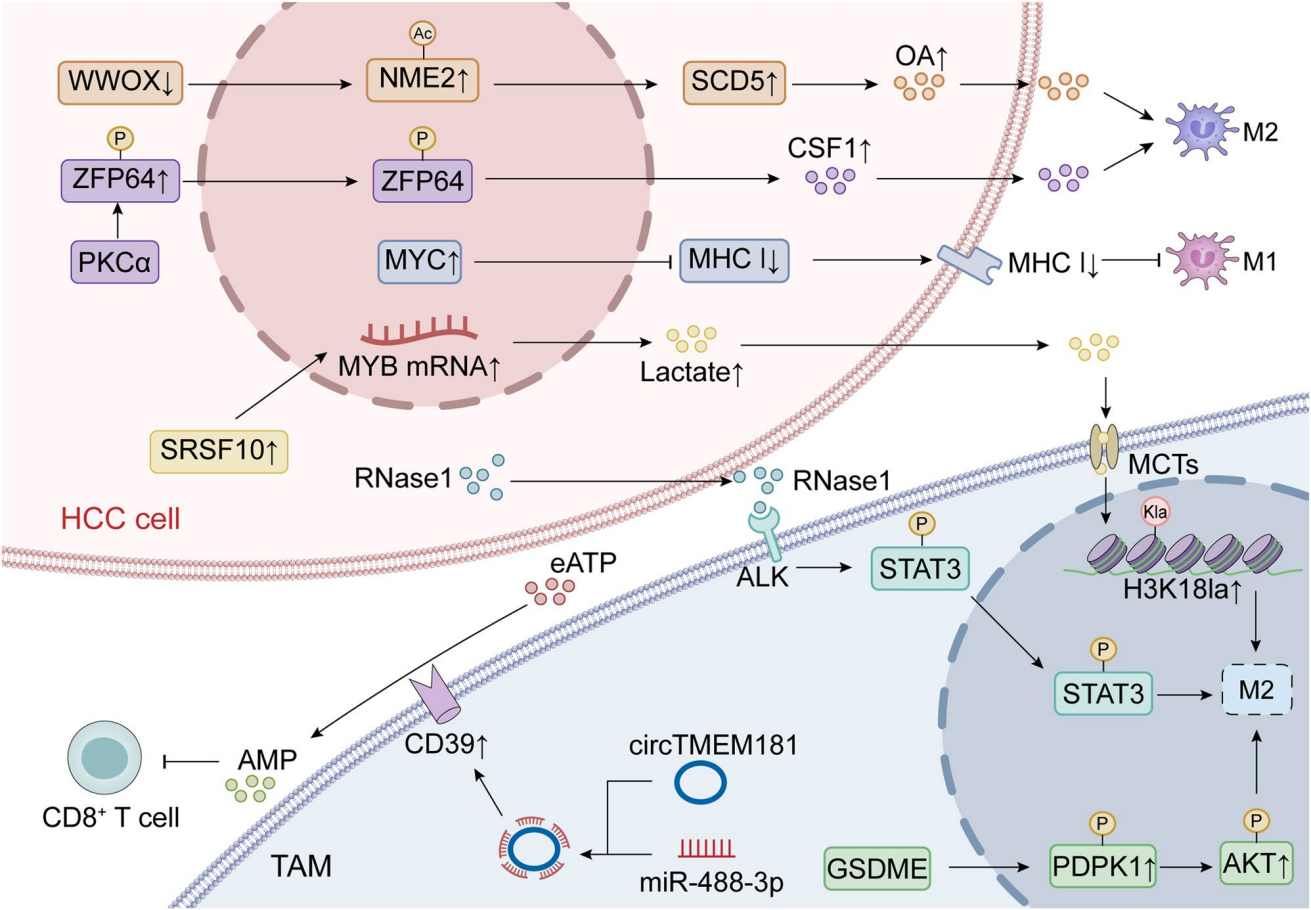
TAMs reprogramming in ICI esistance. TAMs drive ICI resistance in HCC through metabolic reprogramming and transcriptional regulation. Loss of WWOX promotes OA synthesis, inducing TAM polarization to the immunosuppressive M2 phenotype, which impairs CD8^+^ T cell function and weakens PD-1 therapy. Inhibition of SCD5 reprograms TAMs and restores anti-tumor immunity. Overexpression of SRSF10 enhances glycolysis and histone lactylation (H3K18la), further polarizing TAMs to M2. Targeting SRSF10 can improve PD-1 therapy. Additionally, PKCα/ZFP64/CSF1 and MYC signaling drive M2 polarization, contributing to immune suppression and ICI resistance. Combining these pathways with PD-1 or CTLA-4 blockade can enhance therapeutic efficacy of HCC, thus diminishing the overall therapeutic response in HCC

Metabolic reprogramming of TAMs shapes immune suppression and fosters ICI resistance in HCC. WWOX deficiency promotes oleic acid (OA) synthesis via NME2 acetylation and reduced SCD5 binding, polarizing TAMs to an immunosuppressive phenotype that suppresses CD8^+^ T cells and impairs anti-PD-1 therapy. SCD5 inhibition disrupts OA synthesis, reprogramming TAMs and restoring antitumor immunity [182]. Similarly, SRSF10 overexpression creates a glycolysis-histone lactylation feedback loop, stabilizing MYB RNA, increasing lactate production, and promoting M2 macrophage polarization via histone lactylation (H3K18la). This further suppresses interferon-γ^+^ CD8^+^ T cells, enhancing immune evasion. Targeting SRSF10 with the inhibitor 1C8 disrupts this axis, reactivating CD8^+^ T cell activity and improving anti-PD-1 efficacy [109] (Fig. 5).

### Transcriptional regulation of TAM-driven ICI resistance

Zinc finger protein 64 (ZFP64), upregulated in anti-PD-1 resistant HCC, promotes TAM polarization to the immunosuppressive M2 phenotype by activating CSF1 transcription through PKCα-mediated phosphorylation. Targeting the PKCα/ZFP64/CSF1 axis with Gö6976 or lenvatinib restores anti-PD-1 sensitivity by reprogramming the TME [183]. Similarly, MYC overexpression in multiple cancers is linked to immune evasion by increasing immune checkpoint expression and reducing CD8^+^ T cell infiltration. MYC suppresses innate immunity and antigen presentation, impairing T cell activation. Combined PD-L1 and CTLA-4 blockade overcomes this suppression, promoting proinflammatory macrophage recruitment essential for antitumor immunity. Depletion of macrophages abolishes the efficacy of ICI therapy in MYC-driven HCC, indicating that MYC-mediated TAM-driven immune suppression is a critical mechanism of resistance [184] (Fig. 5).

### Other TAM-mediated factors of ICI resistance

In addition, there are several other studies that highlight the role of TAMs in mediating ICI resistance. RNA sequencing of HCC tissues from anti-PD-1 resistant patients revealed upregulation of GSDME, primarily expressed by TAMs, which correlates with poor prognosis. GSDME activates the PI3K-AKT pathway via PDPK1 interaction, promoting M2-like macrophage polarization, reducing CD8^+^ T cell cytotoxicity, and enabling immune evasion. Inhibition of GSDME expression or PDPK1 phosphorylation with Eliprodil shifts TAMs to a less immunosuppressive phenotype and restores T cell activity [185]. Exosomal circTMEM181, elevated in resistant HCC, internalizes into TAMs, sponging miR-488-3p and upregulating CD39, thereby enhancing the adenosine pathway and impairing T cell function [186]. Tumor-secreted RNase1 also induces TAM polarization toward a tumor-promoting phenotype via the ALK signaling pathway, suppressing CD8^+^ T cell activity (Fig. 5). Targeting the RNase1/ALK axis reprograms TAMs, enhances T cell recruitment, and restores anti-tumor immunity. Combined treatment with an ALK inhibitor and anti-PD-1 antibody improves tumor regression and long-term immunity [187].

## Targeting TAMs to overcome ICI resistance in HCC

Given the critical role of TAMs in shaping the TME, targeting TAMs has become a promising strategy to overcome ICI resistance in HCC. An effective approach to target TAMs is by modulating their function and altering their immunosuppressive phenotype. Studies have shown that CD11b agonist can induce a phenotypic shift in TAMs, inhibit NF-κB signaling, and activate the expression of interferon-related genes [122]. This reprogramming restores T cell anti-tumor activity and enhances the overall immune system’s capacity to fight cancer. In addition to targeting TAMs alone, combination immunotherapy strategies are also being widely investigated in clinical trials. Currently, several ongoing trials are evaluating the effectiveness of combining TAM-targeting agents with ICIs (Table 2). In these trials, PLX3397 and similar drugs are being used in combination with PD-1/PD-L1 inhibitors, showing significant clinical potential. These studies not only support the growing evidence for the role of TAMs in immune therapy but also offer new therapeutic options for the personalized treatment of HCC. In summary, the strategy of targeting TAMs in combination with ICIs represents a promising approach to enhancing anti-tumor immunity in HCC.

**Table 2:** Combining TAM-targeting strategies with ICIs for improved HCC treatment

TAM-Targeting Strategy	Immune Checkpoint Inhibitor	Indication	Phase	Outcome Measure	NCT ID
Anti-CSF-1R Antibody (Cabiralizumab)	Anti-PD-1 Antibody	HCC	Phase II	ORR	NCT04050462
Targeting CSF-1R, VEGFR1, -2, -3, TIE2, KIT, RET, RAF-1, BRAF, BRAFV600E, PDGFR, FGFR (Regorafenib)	Anti-PD-1 Antibody (Nivolumab)	HCC	Phase I/II	Incidence of treatment-emergent adverse events (TEAEs), OS, TTP, ORR, PPS, etc.	NCT04170556
Axl/Mer/CSF-1R Selective TKI (Q702)	Anti-PD-1 Antibody (Pembrolizumab)	Advanced esophageal, gastric/GEJ, hepatocellular, and cervical cancers	Phase I/II	Number of participants with TEAEs, tumor response	NCT05438420
CSF-1R Inhibitor (PLX3397)	Anti-PD-1 Antibody (Pembrolizumab)	Advanced melanoma and other solid tumors	Phase I/II	TEAEs, ORR	NCT02452424
CSF-1R Inhibitor (ARRY-382)	Anti-PD-1 Antibody (Pembrolizumab)	Advanced solid tumors	Phase Ib/II	DLT, ORR, DOR, PFS, OS, etc.	NCT02880371
Anti-CSF-1R Antibody (Axatilimab)	Anti-PD-L1 Antibody (Durvalumab)	Solid tumors	Phase I	DLT, MTD, etc.	NCT03238027
CSF-1R Inhibitor (BLZ945)	Anti-PD-1 Antibody (PDR001)	Advanced solid tumors	Phase I/II	Adverse events, Serious adverse events (SAEs), Dose-Limiting Toxicities (DLT), PFS, etc.	NCT02829723
Anti-CSF-1R Antibody (Emactuzumab)	Anti-PD-L1 Antibody (Atezolizumab)	Advanced solid tumors	Phase I	DLTs, MTD, Adverse events, etc.	NCT02323191
Anti-CSF-1R Antibody (LY3022855)	Anti-PD-L1 Antibody (Durvalumab)	Advanced solid tumors	Phase I	MTD, CR, PR, ORR, etc.	NCT02718911
CSF-1R Inhibitor (C-019199)	Anti-PD-1 Antibody (Sintilimab)	Advanced solid tumors	Phase I/II	DLT, ORR, PFS, TEAEs, SAEs, etc.	NCT06220318
CSF-1R Inhibitor (SNDX-6532)	Anti-PD-L1 Antibody (Durvalumab)	Intrahepatic cholangiocarcinoma	Phase II	ORR, OS, PFS, DOR, etc.	NCT04301778
CSF-1R Inhibitor (PEXIDARTINIB)	Anti-PD-L1 Antibody (Durvalumab)	Metastatic/advanced pancreatic or colorectal cancers	Phase I	DLT, ORR, DOR, PFS, Adverse events, etc.	NCT02777710
CSF-1R Inhibitor (IMC-CS4)	Anti-PD-1 Antibody (Pembrolizumab)	Borderline resectable adenocarcinoma of the pancreas	Early Phase I	Number of Patients With a Treatment-related Immunologic Effect, OS, DFS, irORR, etc.	NCT03153410
Agonistic CD40 antibody (RO7009789)	Anti-PD-L1 Antibody (Atezolizumab)	Locally Advanced and/​or Metastatic Solid Tumors	Phase I	Adverse events, SAEs, DLTs, MTD, etc.	NCT02304393
CD40 antibody (CDX-1140)	Anti-PD-1 Antibody (Pembrolizumab)	Advanced malignancies	Phase I	Safety and tolerability of CDX-1140, ORR, etc.	NCT03329950
CCR2/CCR5 antagonist (BMS-813160)	Anti-PD-1 Antibody (Nivolumab)	NSCLC or HCC	Phase II	Major Pathologic Response (MPR), Significant Tumor Necrosis (STN), etc.	NCT04123379

## Toxicity and safety of TAM-targeting therapies

As the combination of CSF-1/CSF-1R inhibitors and ICIs advances into clinical practice, the toxicity profiles of these therapies are becoming increasingly well-defined. When used together in the treatment of solid tumors, these agents exhibit a range of adverse effects (AEs), many of which are class-specific.

One of the most commonly observed toxicities associated with CSF-1R pathway inhibition is the elevation of serum enzymes, such as aspartate aminotransferase (AST), alanine aminotransferase (ALT), creatine phosphokinase (CPK), amylase, lipase, and lactate dehydrogenase (LDH) [188–196]. These increases are usually transient and attributed to impaired hepatic clearance rather than direct organ damage [188]. Specifically, CSF-1R inhibition disrupts the function of Kupffer cells in the liver, resulting in a reduction in enzyme clearance. Preclinical studies support this mechanism, showing that serum enzyme elevations occur without significant histological liver damage [197–199]. However, pexidartinib (PLX3397) is an exception, as it is linked to cholestatic hepatotoxicity, prompting FDA to mandate a Risk Evaluation and Mitigation Strategy (REMS) to address these risks [200]. In addition to liver enzyme elevations, CPK levels are frequently upregulated, particularly with inhibitors like axatilimab [195], emactuzumab [191], pexidartinib [201], vimseltinib [202], and ARRY-382 [196]. Some inhibitors, including LY3022855 [203] and axatilimab [204], are also linked to increased pancreatic enzymes such as amylase and lipase, raising concerns about potential pancreatic toxicity.

Another prominent toxicity of CSF-1/CSF-1R inhibitors is periorbital edema, occurring at high rates with drugs like emactuzumab [205], axatilimab [206], cabiralizumab [192], AMG 820 [190] and LY3022855 [207]. This edema is typically dose-dependent and reversible, with the majority of cases classified as grade 1 or 2 AEs, though some may experience grade 3. The underlying mechanism appears to involve the depletion of LYVE-1⁺ macrophages in the skin, leading to the accumulation of hyaluronic acid (HA) and the activation of matrix metalloproteinases (MMPs) [205].

Hematologic toxicities, particularly anemia, have also emerged as a notable concern in patients receiving CSF-1/CSF-1R inhibitors, especially within combination regimens. Anemia has been observed with several agents, including pexidartinib [208], emactuzumab [191], lacnotuzumab [194], and AMG 820 [190]. Clinical studies have documented instances of grade ≥ 3 anemia, underscoring the importance of vigilant monitoring of hematopoietic function. Gastrointestinal AEs, including nausea, vomiting, anorexia, and diarrhea, are frequently reported with CSF-1R inhibitors such as pexidartinib [208], emactuzumab [191], cabiralizumab [192], LY3022855 [203], and ARRY-382 [196]. In addition, fatigue, a more non-specific toxicity, has been seen with most CSF-1R inhibitors [209]. Skin toxicities, including rash and pruritus, are prevalent with monoclonal antibodies like cabiralizumab [192], emactuzumab [210], lacnotuzumab [194], AMG 820 [190], and LY3022855 [211], with incidence rates ranging from 30 to 70%. These skin reactions are generally mild to moderate in severity and may be accompanied by facial edema or other allergic manifestations.

Overall, the increasing clinical experience with CSF-1/CSF-1R inhibitors reveals a broad spectrum of associated toxicities, necessitating careful monitoring and management to maximize therapeutic benefit. Further studies are needed to clarify the variability in toxicity profiles, understand the potential mechanisms, and assess their impact on therapeutic efficacy, thereby guiding the optimization of personalized treatment strategies.

## Discussion and perspectives

The advent of ICIs has significantly altered the treatment paradigm for HCC, offering new therapeutic possibilities for patients with advanced or unresectable HCC. However, the limited efficacy of ICIs in a substantial proportion of HCC patients underscores the pressing need to address both primary and acquired resistance mechanisms. A growing body of evidence highlights the critical role of TAMs in mediating immune suppression and fostering ICI resistance within the HCC TME. Understanding the intricate interactions between TAMs, immune checkpoints, and the broader immunosuppressive network within the TME is crucial for developing more effective therapeutic strategies.

One of the key challenges in HCC immunotherapy is the predominance of the M2-polarized TAM phenotype, which exerts potent immunosuppressive effects by secreting anti-inflammatory cytokines, promoting angiogenesis, and facilitating EMT [212, 213]. These mechanisms collectively diminish the efficacy of ICIs by blunting T cell responses and reinforcing an immune-cold TME [214]. Therefore, targeting TAMs represents a promising approach to overcoming ICI resistance and enhancing antitumor immunity in HCC. Several strategies have been explored to modulate TAM activity and reprogram their function within the TME. Inhibiting CSF-1R has emerged as a viable approach to depleting TAMs or preventing their M2 polarization [165, 215]. This strategy has demonstrated potential in preclinical and early clinical studies, suggesting that TAM-targeting therapies could synergize with ICIs to reinvigorate antitumor immune responses [190, 216, 217]. Additionally, reprogramming TAMs toward a pro-inflammatory M1 phenotype represents an attractive therapeutic avenue [218]. Agents that enhance macrophage-mediated antigen presentation and increase the secretion of pro-inflammatory cytokines could reshape the immune landscape, making HCC tumors more susceptible to ICIs [219].

Emerging evidence also highlights key signaling pathways and molecular regulators that control TAM polarization and function, which contribute to immune evasion and ICI resistance. For instance, chemokine-mediated mechanisms have been shown to significantly influence TAM behavior. CX3CR1^+^ TAMs, induced by PGE2, promote immune suppression via IL-27 secretion, exacerbating T cell exhaustion and impairing ICI efficacy [180]. Similarly, CCL2 and CCL5 contribute to TAM recruitment and M2 polarization, further undermining ICI response. Targeting the CCL2/CCR2 axis or blocking CCL2/5 with bispecific antibodies has been shown to reverse TAM polarization, shifting them from an M2 phenotype to a pro-inflammatory M1 phenotype, thus improving PD-1 blockade efficacy [13, 127]. Receptor-mediated regulation of TAM function is another critical mechanism of ICI resistance. Receptors such as TREM2 and TREM-1 on TAMs have been implicated in immune suppression within the TME. For example, TREM2^+^ macrophages promote T cell exhaustion and senescence [17], forming an “immune barrier” that impedes the effectiveness of anti-PD-1 therapy. Targeting these receptors with monoclonal antibodies has demonstrated the potential to improve ICI efficacy. Additionally, hypoxia-induced TREM-1 expression in TAMs exacerbates immunosuppression in advanced HCC, impairing T cell cytotoxicity and facilitating tumor progression [172]. Combining TREM-1 inhibition with anti-PD-L1 therapies may be a promising strategy to overcome this barrier. Moreover, metabolic reprogramming of TAMs has been identified as a key factor in ICI resistance. TAMs can shift to a more immunosuppressive phenotype through the dysregulation of metabolic pathways, including glycolysis and fatty acid metabolism. For instance, SRSF10 overexpression drives a glycolysis-histone lactylation feedback loop, promoting M2 polarization and suppressing CD8^+^ T cell responses [109]. WWOX deficiency leads to OA synthesis and M2 macrophage polarization, which inhibits anti-PD-1 efficacy [182]. In addition, transcriptional regulation of TAM-driven immune resistance has become an area of intense focus. ZFP64 is upregulated in anti-PD-1 resistant HCC, promoting TAM polarization towards an immunosuppressive M2 phenotype by activating CSF1 transcription. Inhibiting the PKCα/ZFP64/CSF1 axis has been shown to restore anti-PD-1 sensitivity, highlighting the potential of targeting transcriptional regulators in TAMs to overcome immune resistance [183]. MYC, another key oncogene, contributes to immune evasion by increasing immune checkpoint expression and reducing T cell infiltration [184]. MYC-driven TAM-mediated suppression can be mitigated through combined PD-L1 and CTLA-4 blockade, leading to proinflammatory macrophage recruitment and enhanced antitumor immunity.

Despite these advances, most mechanistic insights are derived from murine models or in vitro studies, which have certain limitations, and several challenges still remain in translating TAM-targeting strategies into clinical practice. The heterogeneity of the HCC TME, the dynamic plasticity of TAM phenotypes, and potential compensatory mechanisms may limit the success of TAM-targeting therapies. For instance, the combination of CSF-1R inhibitors with ICIs has shown disappointing results in clinical trials. A randomized trial assessing cabiralizumab plus nivolumab, with or without chemotherapy, failed to demonstrate any benefit over the control group in pancreatic cancer [220]. Preclinical studies have suggested that the anti-tumor activity of CSF-1R blockade can be counteracted by chemokines-mediated recruitment of polymorphonuclear MDSCs, diminishing the overall efficacy [221]. Also, tumors may compensate by upregulating immunosuppressive cytokines such as IL-4, supporting TAM survival and function even under CSF-1R inhibition [222]. These compensatory mechanisms enable TAM persistance and sustain an immunosuppressive TME, underlining the need to account for such feedback pathways in future therapeutic designs. Recent studies exploring the combination of CSF-1R inhibitors with ICIs and CD40 agonists have shown promise in enhancing anti-tumor immunity [223]. Strategies integrating stereotactic body radiation therapy (SBRT) with nivolumab and cabiralizumab have demonstrated improved responses in patients with advanced solid tumors [224], suggesting a potential synergy between local radiation and TAM-targeting immunotherapy.

To maximize clinical benefit, comprehensive multi-omics approaches, particularly the use of single-cell transcriptomic to uncover TAM heterogeneity [225, 226], will be essential for identifying patient subsets most likely to respond to specific combinatorial regimens. Integration of transcriptomic, proteomic, and spatial profiling data enables the development of reliable predictive biomarkers, facilitating patient stratification and guiding therapeutic decision-making. In addition, advances in nanomedicine are expanding the therapeutic options for cancer immunotherapy, offering novel platforms for the co-delivery of gene-based agents and small-molecule inhibitors [227–229]. The design of TME-responsive nanocarriers, capable of site-specific activation under acidic, hypoxic, or reductive conditions, allows for the spatiotemporally controlled release of therapeutic payloads. Such targeted delivery systems enhance intratumoral drug accumulation, potentiate antitumor immune responses, and minimize systemic toxicity. Studies have shown that co-delivery of CSF-1R inhibitors with cytokine IL-12 or paclitaxel can remodel the immunosuppressive TME, prompt T cell mediated immunity, and effectively suppress tumor growth and metastasis [228, 229].

In future, a deeper understanding of the interplay between TAMs and other immune components within the TME will be instrumental in designing next-generation immunotherapeutic strategies. Rational combinations that integrate ICIs with TAM-modulating agents, anti-angiogenic therapies, and metabolic interventions hold great promise for overcoming immune resistance in HCC. Continued preclinical and clinical investigations are needed to refine these strategies and translate mechanistic insights into therapeutic benefits for patients.

## Conclusion

In conclusion, this review explored the role of TAMs in HCC, emphasizing their critical involvement in immune evasion and resistance to ICIs. TAMs exhibit significant heterogeneity, predominantly polarized into M2 phenotype that drives tumor progression, immune suppression, angiogenesis, and metastasis. We discussed the complex signaling pathways governing TAM polarization and the various mechanisms by which TAMs promote an immunosuppressive microenvironment in HCC. In particular, M2-polarized TAMs were shown to play a central role in facilitating ICI resistance by inhibiting T cell activation and promoting tumor immune evasion. Although targeting TAMs by depleting their population or reprogramming them to a pro-inflammatory M1 phenotype shows therapeutic promise, significant challenges remain in translating these strategies into clinical practice. The heterogeneity of the HCC TME, dynamic TAM plasticity, and compensatory mechanisms hinder the success of TAM-targeting therapies, as evidenced by the disappointing results of CSF-1R inhibitor combinations with ICIs in clinical trials. Further investigation into TAM modulation, combined with emerging multi-omics approaches to reveal TAM heterogeneity, will be crucial for overcoming ICI resistance in HCC. The integration of novel therapeutic strategies, such as nanomedicine and TME-responsive drug delivery systems, offers exciting potential to enhance the efficacy of TAM-targeting therapies and improving patient outcomes.

## Data Availability

No datasets were generated or analysed during the current study.

## Abbreviations

HCC: Hepatocellular carcinoma
ICIs: Immune checkpoint inhibitors
TAMs: Tumor-associated macrophages
HBV: Hepatitis B
HCV: Hepatitis C
NAFLD: Non-alcoholic fatty liver disease
TACE: Transarterial chemoembolization
TME: Tumor microenvironment
EMT: Epithelial-mesenchymal transition
CSF-1R: Colony-stimulating factor 1 receptor
LAG-3: Lymphocyte activation gene-3
TIM-3: T cell immunoglobulin and mucin-domain containing-3
TIGIT: T-cell immunoreceptor with Ig and ITIM domains
NK: Natural killer
VISTA: V-domain Ig suppressor of T cell activation
APCs: Antigen-presenting cells
ADCC: Antibody-dependent cell-mediated cytotoxicity
TMB: Tumor mutational burden
CTLs: Cytotoxic T lymphocytes
Tregs: Regulatory T cells
MDSCs: Myeloid-derived suppressor cells
Gal1: Galectin-1
LTA4H: Leukotriene A4 hydrolase
PLA2G7: Macrophage-specific phospholipase A2 Group VII
MCTCs: Macrophage-coated tumor clusters
mtDNA: Mitochondrial DNA
FAO: Fatty acid oxidation
TIPE1: Tumor necrosis factor α-induced protein 8-like 1
TREM1: Triggering receptor expressed on myeloid cells 1
PUM1: Pumilio RNA-binding family member 1
TFRC: Transferrin receptor
RIPK3: Receptor-interacting protein kinase 3
ROS: Reactive oxygen species
miRNAs: MicroRNAs
Hh: Hedgehog
GAS5: Growth arrest-specific 5
scRNA-seq: Single-cell RNA sequencing
FoxO1: Forkhead box O1
CSF-1: Colony-stimulating factor-1
CX3CR1: CX3C motif chemokine receptor 1
PGE2: Prostaglandin E2
SOX18: SYR-Related High-Mobility Group Box 18
MRD: Minimal residual disease
OA: Oleic acid
ZFP64: Zinc finger protein 64
ctDNA: Circulating tumor DNA
CTCs: Circulating tumor cells
NIK: NF-κB-inducing kinase
TANs: Tumor-associated neutrophils
CAFs: Cancer-associated fibroblasts
AEs: Adverse effects
AST: Aspartate aminotransferase
ALT: Alanine aminotransferase
CPK: Creatine phosphokinase
LDH: Lactate dehydrogenase
REMS: Risk Evaluation and Mitigation Strategy
HA: Hyaluronic acid
MMPs: Matrix metalloproteinases
SBRT: Stereotactic body radiation therapy
ORR: Objective Response Rate
TEAEs: Treatment-emergent adverse events
SAEs: Serious adverse events
DLT: Dose-Limiting Toxicities
MPR: Major Pathologic Response
STN: Significant Tumor Necrosis
